# Migratory history of the threespine stickleback *Gasterosteus aculeatus* in western Ireland

**DOI:** 10.1016/j.heliyon.2024.e28425

**Published:** 2024-03-22

**Authors:** Takaomi Arai, Daisuke Ueno, T. Kieran McCarthy

**Affiliations:** a Environmental and Life Sciences Programme, Faculty of Science, Universiti Brunei Darussalam, Jalan Tungku Link, Gadong, BE 1410, Brunei Darussalam; b Graduate School of Fisheries Sciences, Kitasato University, 160-4 Sanriku, Ofunato, Iwate 022-0101, Japan; c Department of Zoology, National University of Ireland, Galway, University Road, Galway, Ireland

**Keywords:** Diverse migration, Element, Environmental history, Life history, Otolith microchemical fingerprints

## Abstract

Microchemical analysis of trace elements in otoliths and bio-mineralised earstones of teleost fishes is an emerging approach to analysing the environmental migratoryand life histories of fish species. The migration history of the three-spine stickleback (*Gasterosteus aculeatus*) collected in western Ireland was examined using calcium (Ca) and strontium (Sr) concentrations in otoliths. The otolith Sr:Ca values fluctuated with the habitat. The habitat use and migration history of *G. aculeatus* can be categorised into two types, as determined by the mean value and life history transect of the otolith Sr:Ca; that is, freshwater and estuarine residents, whereas there were no anadromous sticklebacks which is believed to be a typical migration pattern in the species. The otolith Sr:Ca profiles of the freshwater resident fishes exhibited constantly low Sr:Ca values, averaging 0.41–0.58 × 10^−3^ from the core towards the edge. However, the otolith Sr:Ca profiles of the estuarine resident fishes exhibited constantly high Sr:Ca values from the core towards the edge without a clear transition point from low to high Sr:Ca values, as found in the anadromous fish, averaging 1.82–4.26 × 10^−3^. The present study is the first published confirmation that 100 % of sticklebacks living in coastal habitats in Ireland > have an estuarine resident migratory pattern, constantly residing in marine environments or brackish water throughout their lifespan and not in freshwater environments in Ireland.

## 1 Introduction

In the inner ear of teleosts, the otolith, located in a membranous labyrinth, is a useful research material from which to obtain information about the migration and life history over the course of the lifespan of a single fish [1,2]. Due to the fact that Sr and Ca have similar ionic radii, the two elements interchange in the otolith aragonite lattice [3]. Otolith Sr:Ca values have been shown to be higher in marine and estuarine environments than in freshwater environment [2]. Therefore, the Sr:Ca values obtained from otoliths could be a reliable tool for reconstructing the migration history and habitat use of diadromous fish migrating between marine or brackish waters and freshwater.

Otolith microchemistry of the three-spine stickleback (*Gasterosteus aculeatus*) has revealed diverse migrations in aquatic environment [[4], [5], [6]]. Opportunistic anadromous migration occurring in a new estuarine resident migration pattern has been revealed using otolith Sr:Ca fingerprints in Japan [[4], [5], [6], [7], [8]]. Recently, Arai et al. [6] confirmed that Sr and Ca concentrations and the corresponding Sr-to-Ca ratio in the otoliths of the three-spine stickleback increased with increasing salinity in environmental waters. Therefore, otolith microchemical fingerprints are reliable tools for reconstructing the migration histories and habitat preferences of *G. aculeatus.* The otolith Sr:Ca fingerprints have also been used to elucidate a new migratory pattern in another stickleback species, the ninespine stickleback (*Pungitius pungitius*). Brackish water and freshwater resident life histories are two general life history types; however, an alternative anadromous life history has been identified based on otolith microchemical fingerprints [[9], [10], [11]]. Despite these advances in otolith analysis, the details of habitat use, migration history, and their relative composition throughout the wide distribution range of three-spine sticklebacks are unclear.

*Gasterosteus aculeatus* is a small teleost fish (50–100 mm) broadly distributed throughout the Northern Hemisphere [12] and found in various aquatic environments, such as marine water, estuarine water, and freshwater habitats. Many populations are annual, and the fish is believed to have a short lifespan [12]. The life history and migration patterns of the species have been categorised as two basic types: freshwater resident type and an anadromous type [12]. However, Arai et al. [[4], [5], [6], [7]], through analysis of otolith microchemical fingerprints, discovered that some fish show a new migration type, estuarine residents which spend their whole life history in marine, brackish and/or estuarine environments close to but not within natural freshwater environments. Therefore, the life history and migration of three-spine sticklebacks are complex and variable. Information regarding the migration history of this species remains at a rudimentary level, which prompted the more thorough analysis described in this study.

We examined the otolith microchemistry of three-spine sticklebacks collected in western Ireland. The otolith microchemical fingerprints yield information on the migratory history and habitat use characteristics of *G. aculeatus* as well as elucidating the diversity of migration patterns between freshwater and marine environments.

## 2 Materials and methods

We followed the ethical guidelines for animal use at the Universiti Brunei Darussalam (UBD), and our protocols were approved by the animal ethics committee at UBD (Approval Code: UBD/RSCH/1.4/FICBF(b)/2021/037; Approval Date: September 15, 2021).

**Fig. 1:**
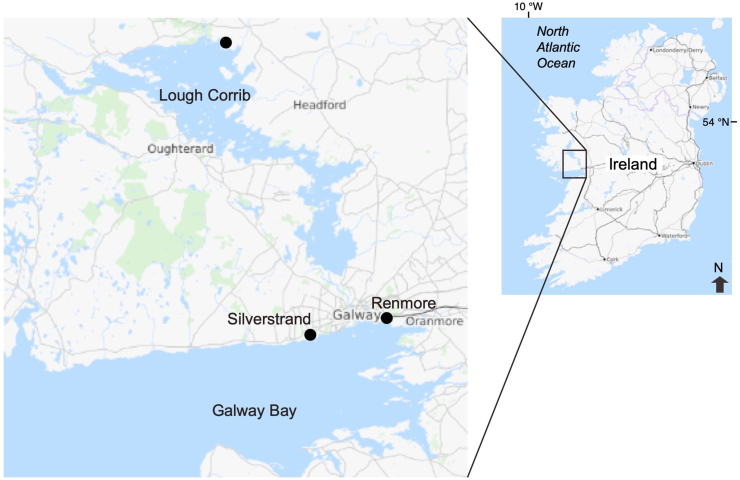
Sampling sites for the threespine stickleback (*Gasterosteus aculeatus*) collected in western Ireland. The black dots indicate the sampling sites of the specimens. The base map was downloaded from the OpenStreetMap (open access) at https://www.openstreetmap.org ((Data© OpenStreetMap contributors).

*Gasterosteus aculeatus* specimens (30 total) were collected using dip nets from brackish (marine) and freshwater environments in western Ireland from March to June 2008 (Table 1, Fig. 1). Ten individual sticklebacks from freshwater habitats were sampled at Lough Corrib; the sampling site was located above the intertidal area. Twenty sticklebacks from brackish and seawater habitats were collected from the beaches of Renmore (10 specimens) and Silverstrand (10 specimens), facing Galway Bay (Table 1, Fig. 1). These locations are intertidal zones influenced by tidal flow. The sagittal otoliths were removed from each fish after the measurement of total length (TL) (Table 1). All the otoliths were embedded in epoxy resin (Epofix; Struers, Copenhagen, Denmark) and placed on glass slides. Each otolith was ground to expose its core using a grinding machine (Discoplan-TS; Struers, Copenhagen, Denmark). Thereafter, the otoliths were polished using an automated polishing wheel (PdM-Force-20, Struers) with an oxide polishing suspension. After grinding and polishing, each otolith was cleaned and rinsed with deionised water prior to the Ca and Sr analyses.

**Table 1:**
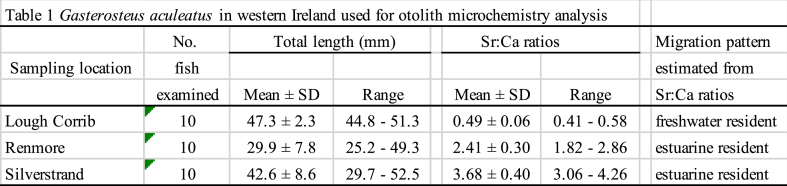
*Gasterosteus aculeatus* in western Irel and used for otolith microchemistry analysis.

Each otolith was coated with platinum (Pt)-palladium (Pd) using a high- vacuum evaporator for electron microprobe analysis. Each otolith was analysed for Ca and Sr concentrations for life-history transect analysis, measured along a line down the longest axis of each otolith from the core towards the edge using a wavelength dispersive X-ray electron microprobe (JEOL JXA-8900R, Jeol, Tokyo, Japan). The electron beam was focused on a point 2 μm in diameter and was measured at 2 μm intervals. The accelerating voltage and beam current were 15 kV and 1.2 × 10^−8^ A, respectively. Wollastonite (CaSiO_3_) and Tausonite (SrTiO_3_) were used as standards.

Comparisons of average Sr:Ca values in the three groups (locations) were performed using a Kruskal-Wallis test, and comparisons of average Sr:Ca values between the two groups were performed using *post hoc* Mann-Whitney *U* tests. Sr:Ca values at the edge of otoliths incorporate the most recent life history information of the fish just before capture or natural death, and have been utilised to reconstruct habitat use [6]. The Sr:Ca values at the otolith edges were examined in all specimens. The Sr:Ca values at the otolith edge were examined using one-way analysis of variance (ANOVA) with Tukey's *post hoc* test for multiple comparisons.

## 3 Results

**Fig. 2:**
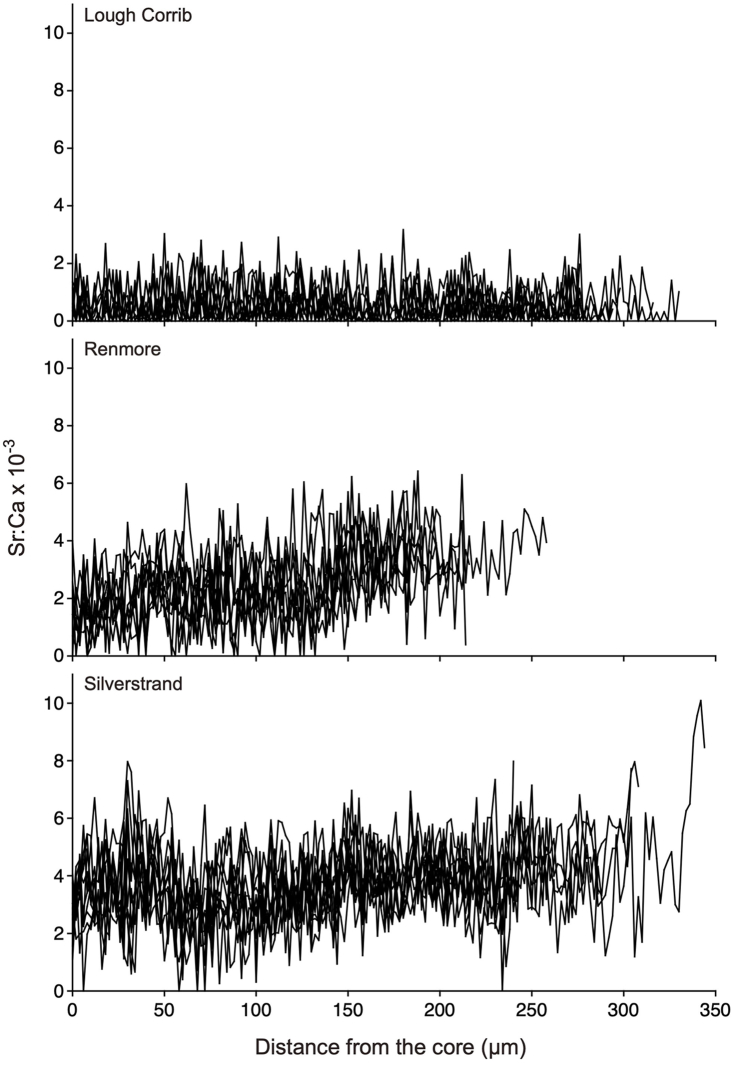
Migratory history of *G. aculeatus* as indicated by the temporal pattern of otolith Sr:Ca values from specimens collected in western Ireland.

Sr:Ca measured along a transect from the core towards the otolith margin from the Lough Corrib showed consistently low values, averaging 0.49 × 10^−3^ ± 0.06 × 10^−3^ (±SD) (range: 0.41–0.58 × 10^−3^) (Table 1, Fig. 2), suggesting continued habitation in a freshwater environment from hatching. The Sr:Ca values in the otoliths from Renmore and Silverstrand exhibited constantly high values around the core towards the edge, averaging 2.41 × 10^−3^ ± 0.30 × 10^−3^ (range: 1.82–2.86 × 10^−3^) and 3.68 × 10^−3^ ± 0.40 × 10^−3^ (range: 3.06–4.26 × 10^−3^), respectively (Table 1 and Fig. 2). Significant differences were found in the average otolith Sr:Ca values among the three locations (Kruskal-Wallis test, H = 25.81, p < 0.0001), and there were significant differences in all combinations (Mann Whitney-*U* test, U = 9–16, p < 0.0001). The Sr:Ca ratios at the otolith edges at the three sites ranged from 0.0 to 10.09 × 10^−3^. Significant differences were observed for all the combinations (ANOVA, df = 2, F = 90.440, p < 0.0001). Sr:Ca values in otolith from Renmore were highest, averaging 6.06 × 10^−3^ ± 2.13 × 10^−3^ (p < 0.0001); those from Silverstrand were second higher, averaging 3.33 × 10^−3^ ± 1.22 × 10^−3^ (p < 0.0001); and those from the Lough Corrib was lowest, averaging 0.55 × 10^−3^ ± 0.68 × 10^−3^ (p < 0.0001).

## 4 Discussion

**Fig. 3:**
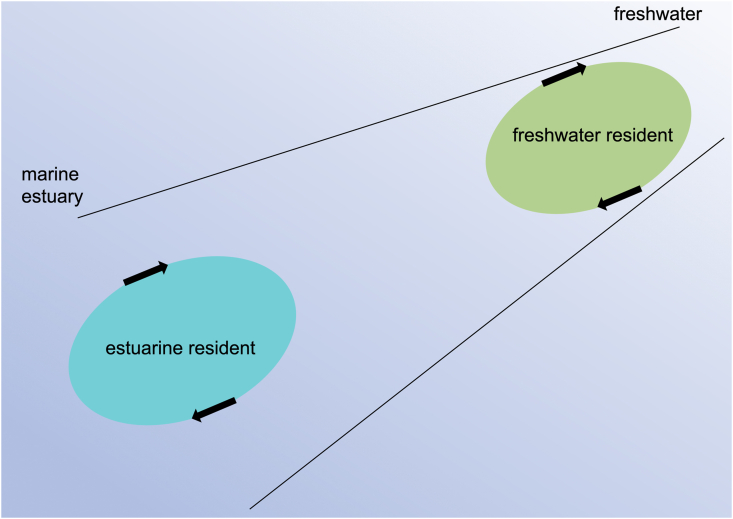
Migratory histories of *G. aculeatus* populations in Ireland, as determined by otolith microchemical fingerprints. The migration patterns of *G. aculeatus* were determined to be either freshwater or estuarine residents.

Based on the mean values of *G. aculeatus* otolith Sr:Ca and life history transects (Table 1, Fig. 2), the habitat use and migration history of the species in the western Ireland can be categorised as either estuarine resident or freshwater resident (Fig. 3). In the Renmore and Silverstrand specimens collected from coastal beach areas, consistently high Sr:Ca values were found from the core towards the edge of the otolith. These fish lived their entire lives, from day of hatching on, in marine water and/or brackish water. Based on stickleback specimens in Japan, the estuarine resident life history was classified as constantly high Sr:Ca values from the core towards the edge of the otolith without a shift from a low phase to a high phase [4]. In the present study we also found that estuarine residents had a crucial migration pattern on the western coast of Ireland. *G. aculeatus* is generally divided into freshwater (fluvial) and anadromous types according to its lifestyle [12]. Based on its external morphological characteristics, *G. aculeatus* can be differentiated as either anadromous or freshwater resident [13,14]. Freshwater sticklebacks have a small number of plates (low-plate morph), whereas the anadromous *G. aculeatus* has a completely plated morph [13,14]. A bimodal size distribution was found to coexist in a habitat, and the average size was smaller in freshwater residents than in anadromous specimens [[15], [16], [17]]. However, morphological characteristics can only be used in restricted areas because of local variations in features [15,16]. The present results did not show an anadromous migration pattern using the otolith Sr:Ca signatures (Fig. 2, Fig. 3). The mean otolith Sr:Ca ratios were significantly different in the coastal habitats of Renmore and Silverstrand, and this discrepancy may be due to differences in habitat salinity between the two sites. The otolith Sr:Ca in *G. aculeatus* is positively correlated with environmental salinity; hence, the ratio is applicable as a tracer to reconstruct migration history throughout life [6]. A similar diversity of migration and a new migration type have also been found in the ninespine stickleback, *P. pungitius* by means of otolith Sr:Ca signatures [[9], [10], [11]]. This species is known to have an alternative anadromous migration pattern, together with two typical life history types: brackish water and freshwater resident migration histories. The anadromous life history which has been believed to be based on morphological characteristics, is not necessarily consistent with the migration pattern.

The mean and edge otolith Sr:Ca values suggest that the Lough Corrib specimens had constantly lived in freshwater environments. Interestingly, a discrepancy in the edge Sr:Ca ratios in the otolith was found between the Renmore and Silverstrand specimens. Sr:Ca values in the otolith edge from Renmore were higher than 6.0 × 10^−3^, which suggests that these fish might have lived in the full range of the marine environment just before capture. The specimens in Silverstrand showed approximately 3.0 × 10^−3^ in otolith Sr:Ca values at the otolith edge. These results suggest that the specimens in Silverstrand might live in less saline waters, such as estuaries or brackish water habitats, before sampling.

*Gasterosteus nipponicus* which was transferred from the Japan Sea form of *G. aculeatus* [18], was found to spawn in marine tidal pools in northern Japan (Hokkaido Island) [8]. All tidal pools were almost completely saline, and both males and females were collected from the pools. *G. aculeatus* collected from marine tidal pools showed constantly high otolith Sr:Ca values from the core towards the edge [8]. Therefore, *G. aculeatus* in tidal pools could reside in full-salinity environments throughout their lives during the egg, newly hatched juvenile, juvenile, adult, and maturation periods. Our findings and those of previous studies suggest that the majority of *G. aculeatus* reside in intertidal waters, such as estuaries, salt marshes, beaches, and tidal pools, and can be estuarine residents that live in marine water and/or brackish water environments without freshwater growth.

Estuarine residents might have more advantages in life history strategy than freshwater residents and anadromous in *G. aculeatus*. In anadromous sticklebacks, migration from freshwater to marine habitats may be energetically costly because of increased swimming activity. There may also be a physiological cost in accordance with osmotic regulation for migration between different salinity environments. In contrast, estuarine residents may benefit from continuous residence in environments with low fluctuations in salinity. Coastal habitats are more productive than freshwater habitats in terms of survival and growth. Primary production is higher in marine habitats at higher latitudes than in freshwater habitats [19], where *G. aculeatus* is distributed. Growth and survival may be enhanced in estuarine residents living in productive and suitable habitats. Further studies on otolith Sr:Ca ratios in various habitats throughout the distribution range are needed. To elucidate the diverse migration patterns in *G. aculeatus*, the mean otolith Sr:Ca, as an indicator of environmental habitat use and migration patterns, should be compared among habitats.

## 5 Conclusion

The global distribution of three-spine stickleback ranges across the Northern Hemisphere. To date, otolith studies of this species have only been carried out with specimens from coastal waters of Japan [[4], [5], [6], [7], [8]]. The present study is the first in which the migration and life history of three-spine stickleback has been investigated in Irish waters. The results revealed two migration patterns for freshwater and estuarine residents (Fig. 3), whereas no specimen was found with a typical anadromous migration pattern. Our findings suggest that the classical method of morphological observation is not useful for identifying species migration or life history patterns. The otolith Sr:Ca of the three-spine stickleback reflects the environmental salinity of each habitat. Our results confirmed that the otolith Sr:Ca is a reliable indicator for categorising migration patterns in three-spine sticklebacks. The three-spine stickleback in Irish waters showed diverse migration patterns in environmental waters based on otolith microchemical fingerprints. Given that this type of analysis has only been performed on specimens from Japanese and—with this study—Irish populations, further analyses, using otolith microchemical fingerprints from various populations are needed in order to generalise the migration history and habitat use of the species throughout its distribution range.

## Data availability statement

Data will be made available on request.

## CRediT authorship contribution statement

**Takaomi Arai:** Writing – review & editing, Writing – original draft, Visualization, Validation, Supervision, Software, Resources, Project administration, Methodology, Investigation, Funding acquisition, Formal analysis, Data curation, Conceptualization. **Daisuke Ueno:** Methodology, Investigation, Data curation. **T. Kieran McCarthy:** Methodology, Investigation.

## Declaration of competing interest

The authors declare that they have no known competing financial interests or personal relationships that could have appeared to influence the work reported in this paper.
